# Does human homology reduce the potential immunogenicity of non-antibody scaffolds?

**DOI:** 10.3389/fimmu.2023.1215939

**Published:** 2023-11-07

**Authors:** Anne S. De Groot, Sundos Khan, Aimee E. Mattei, Sandra Lelias, William D. Martin

**Affiliations:** ^1^ EpiVax, Providence, RI, United States; ^2^ University of Georgia, Center for Vaccines and Immunology, Athens, GA, United States

**Keywords:** Tregitope, JanusMatrix, tolerance, immunogenicity, protein therapeutics, non-antibody scaffold proteins

## Abstract

Biologics developers are moving beyond antibodies for delivery of a wide range of therapeutic interventions. These non-antibody modalities are often based on ‘natural’ protein scaffolds that are modified to deliver bioactive sequences. Both human-derived and non-human-sourced scaffold proteins have been developed. New types of “non-antibody” scaffolds are still being discovered, as they offer attractive alternatives to monoclonals due to their smaller size, improved stability, and ease of synthesis. They are believed to have low immunogenic potential. However, while several human-sourced protein scaffolds have not been immunogenic in clinical studies, this may not predict their overall performance in other therapeutic applications. A preliminary evaluation of their potential for immunogenicity is warranted. Immunogenicity risk potential has been clearly linked to the presence of T “helper” epitopes in the sequence of biologic therapeutics. In addition, tolerogenic epitopes are present in some human proteins and may decrease their immunogenic potential. While the detailed sequences of many non-antibody scaffold therapeutic candidates remain unpublished, their backbone sequences are available for review and analysis. We assessed 12 example non-antibody scaffold backbone sequences using our epitope-mapping tools (EpiMatrix) for this perspective. Based on EpiMatrix scoring, their HLA DRB1-restricted T cell epitope content appears to be lower than the average protein, and sequences that may act as tolerogenic epitopes are present in selected human-derived scaffolds. Assessing the potential immunogenicity of scaffold proteins regarding self and non-self T cell epitopes may be of use for drug developers and clinicians, as these exciting new non-antibody molecules begin to emerge from the preclinical pipeline into clinical use.

## Introduction

**Figure 1:**
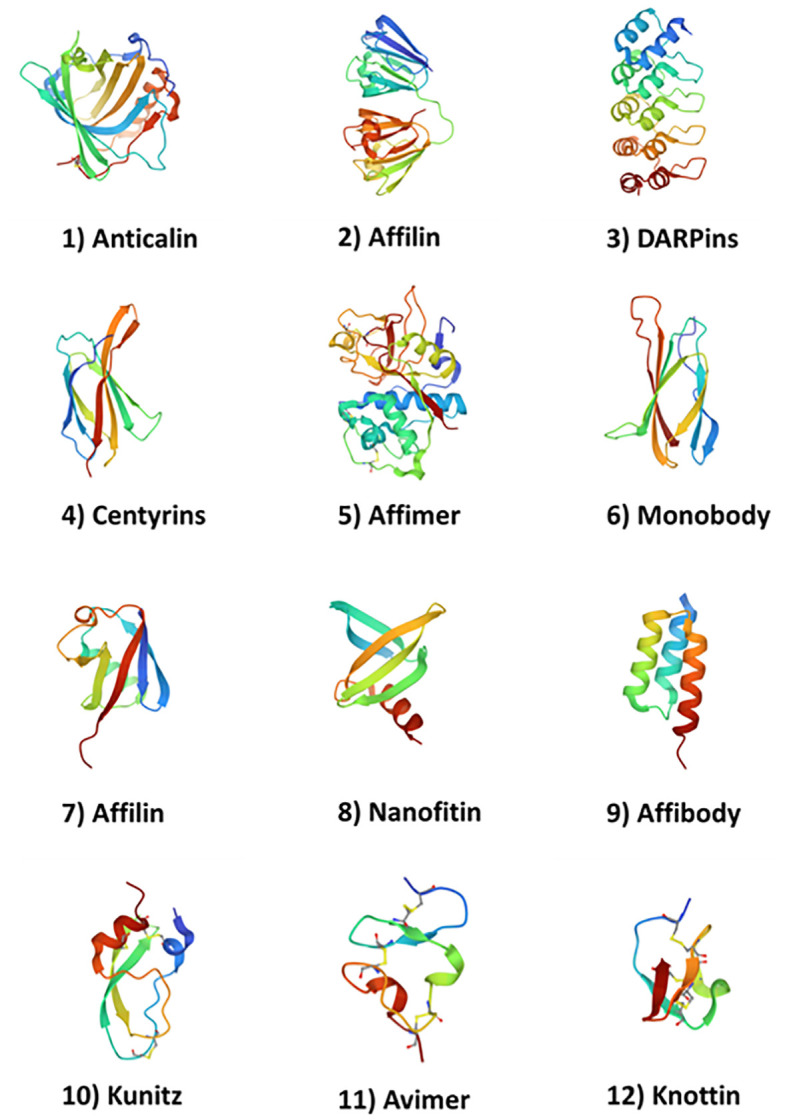
Structures of 12 example protein scaffolds. (1) Anticalins (PDB ID: 4GH7); (2) Affilin (g-B-crystallin based) (PDB ID: 2JDG); (3) DARPins (PDB ID: 1MJ0); (4) Centyrins (PDB ID: 5L2H); (5) Affimer (PDB ID: 1NB5); (6) Adnectin/Monobody (PDB ID: 1TTG); (7) Affilin (ubiquitin based) (PDB ID: 1UBI); (8) Nanofitin (PDB ID: 4CJ2); (9) Affibody (PDB ID: 3MZW); (10) Kunitz (PDB ID: 1KTH); (11) Avimer (PDB ID: 1AJJ); and (12) Knottin (PDB ID: 2IT7. Structures were obtained from the PDB (https://www.rcsb.org/ on April 26^th^, 2023).

Non-antibody molecules known as engineered scaffold proteins emerged in the early 2000’s (see Gebauer and Skerra (1) for a review of the field). While they are currently used for a number of diagnostics and proteomics applications (2), these novel protein drugs provide developers with an attractive alternative to antibodies. They are often derived from repeating domains of natural human proteins (or highly structured non-human proteins) and feature loops that resemble the CDR domains of monoclonal antibodies that can be modified to express targeting sequences. Examples include Anticalins (from lipocalin) (3, 4); DARPins (from Ankyrin) (5); Affilin (from ubiquitin or gamma-B-crystallin) (6); derivatives of Fibronectin such as Monobodies (7) and Adnectins (8, 9); Avimers (10) and Kunitz scaffold proteins (11, 12). Non-human derived scaffold proteins include Affibodies (from protein A of Staphylococcus) (13) and Nanofitins (14). Knottins (15) are derived from eukaryotic organisms but are also present in several plant species. These example non-antibody scaffolds with their source proteins are described in **Table 1** and the protein backbone representations of twelve example protein scaffolds are illustrated in **Figure 1**.

**Table 1:** Types of non-antibody-based scaffold proteins.

COMMON NAME	SOURCE ORGANISM	PROTEIN	AA LENGTH	REF.
**Anticalin**	Human	Lipocalin	178	(3, 4)
**Affilin**	Human	Gamma-B-Crystallin	175	(6)
**DARPin**	Human	Ankyrin repeat proteins	166	(5)
**Centyrin**	Human	Fibronectin Type III domain	103	(5)
**Affimer**	Human	Cystatin-A	98	(8)
**Adnectin**	Human	Fibronectin	94	(7, 9)
**Affilin**	Human	Ubiquitin	76	(6)
**Nanofitin/affitin**	Bacteria	DNA-Binding Sac7d	65	(14)
**Affibody**	Bacteria	IgG-binding protein A	58	(13)
**Kunitz**	Human	Collagen Alpha-3 VI-Chain	58	(11, 12)
**Avimer**	Human	Low-density lipoprotein receptor	37	(10)
**Knottin**	Plant	Trypsin Inhibitor 2	28	(15)

These proteins have attractive features such as stability in circulation, ease of modification, developability and relatively low immunogenicity in preclinical animal models, but their true potential for immunogenicity in clinical applications is not well understood. The number of (HLA DRB1) T helper epitopes that may be present, to drive Anti-Drug-Antibody (ADA) responses has not been quantified and reported. More specifically, modifications to the scaffold to improve affinity for target ligands may introduce new T cell epitopes, contributing to effector (inflammatory) T cell response and ADA. Furthermore, whether or not natural T regulatory epitope sequences (similar to IgG “Tregitopes” previously identified by our group (16) and others (17)) are present in the sequences of scaffold proteins, has yet to be well-defined. These important self-regulatory epitopes may modulate or suppress immunogenic potential of these novel scaffolds.

While many protein scaffold-based therapeutics are in development, only a few have been approved or have reached the phase III clinical stage of development (18, 19), thus it is difficult to determine whether scaffolds are truly advantageous, compared to monoclonal antibodies, in terms of their relative potential for immunogenicity. Nevertheless, it is worth evaluating their potential for immunogenicity using epitope-mapping tools currently used in the industry. Here we used tools that are available in the proprietary ISPRI (Immunogenicity Screening and Protein Re-engineering) toolkit. A similar approach can be performed using publicly-available tools, namely epitope mapping, epitope cluster identification, and ranking with respect to epitope density corrected for potentially tolerogenic epitopes.

Using ISPRI, we assessed the twelve example non-antibody scaffold backbone sequences for this discussion and will focus on their potential for immunogenicity (while understanding that we are not describing the exact immunogenic potential of any specific non-antibody scaffold drug product). While additional studies are clearly needed, this immunoinformatics perspective may provide an initial guide to the assessment of immunogenic potential of scaffold proteins, which might begin with in silico analysis of HLA DRB1-restricted T cell epitope content and a search for potentially tolerogenic epitopes.

### Methods for evaluating the immunogenicity risk of scaffold proteins

Both in silico and *in vitro* methods for evaluating the potential immunogenicity of biologic proteins have been developed and deployed over the past 15 years. It is now well known that fully human-derived biologic products such as Erythropoietin, Thrombopoietin and monoclonal antibodies have the potential to induce unwanted and unexpected immune response (20, 21). Over the years, multiple human-derived biologics were identified as being immunogenic in clinical studies (22).

Drug developers are using both in silico and *in vitro* methods to define and de-risk inflammatory T helper epitopes in therapeutic proteins prior to advancing candidates to the clinic (see Jawa et al. for a review of current methods (23)). In silico tools that resemble the tools used in this analysis include several, that are available on public websites (24); these can be integrated into internal pipelines. Large and mid-sized companies tend to use secure, web-based commercial platforms such as the ISPRI toolkit developed by De Groot and Martin to assess immunogenicity risk and de-risk monoclonals (25–32), or their own, in house-developed tools. These types of toolkits offer epitope-mapping algorithms such as EpiMatrix (33), as well as other tools, to rank biologic candidates and to compare them with biologics for which the immunogenicity risk profile has been well-established.

T cell epitope-mapping tools are always at the core of immunogenicity risk assessments that are performed using in silico toolkits. ISPRI uses EpiMatrix, a matrix-based algorithm, to define linear nine-to-ten amino acid long peptides that are predicted to bind to human leukocyte antigen (HLA) molecules [also called major histocompatibility complex (MHC)], where they are presented to T cell receptors (TCR) of T cells (CD4+ and CD8+). Since the sequence of T cell epitopes are linear, EpiMatrix uses a set of proprietary matrices to score each amino acid in each nine-mer (or 10 mer) sequence for matches (positive or negative) to well-defined amino acid matrices of possibilities. The final score reflects the probability that a given peptide will bind to a specific MHC (HLA class I or class II) molecule. These scores are normalized across alleles, enabling the comparison of epitopes across proteins and the ranking and comparison of proteins by total T cell epitope content per 1000 9-mer frame assessments, on a normalized immunogenicity scale (see **Supplemental Figure 1** and **Supplemental Information** for a more detailed description of the in silico methods applied to this analysis). Additional features such as the accuracy of the tools (88% for HLA DRB1 alleles, overall) and examples of their use are described in a review of immunoinformatics tools by De Groot et al., 2020 (33).

Of the different types of epitopes that may induce ADA, HLA DRB1-restricted class II epitopes are the most common on antigen-presenting cells. Concentrated predicted HLA DRB1 T cell epitope ‘regions or clusters’ are easily identified in protein sequences (**Supplemental Figure 2**). These clusters are often found in highly immunogenic vaccine antigens and tend to be associated with immunogenicity when they are identified in protein therapeutics. Once identified, T cell epitope clusters can be compared to sequences of previously published epitopes [and MAPPS data (34)] by searching for homologous MHC-eluted sequences in immune epitope databases such as IEDB (35). The MAPPS approach involves identification of peptides eluted from MHC molecules, followed by mass spectrometry-based analysis. The availability of MAPPS data in online databases has improved the rapid validation of computationally predicted T cell epitopes, as in silico results can be compared to the extensive list of peptides eluted from MHC that is stored online (34). However, it is important to note that while MAPPS assays identify MHC ligands, it does not determine whether the ligands are immunogenic or tolerogenic T-cell epitopes.

### Integrating assessment of tolerogenic epitopes

The presence of a T cell epitope does not guarantee that a T helper cell will be activated. In fact, a subset of T cells, called regulatory T cells (Tregs) that are responsible for maintaining immune tolerance and preventing autoimmune diseases can also recognize T cell epitopes in self-proteins such as scaffold proteins. Tregs act as a brake on the immune response by suppressing the activity of other immune cells, including T effector cells that might drive antibody responses (ADA). Tregs are present in the B cell follicle where they control the maturation (and increasing affinity) of antibodies and the development of memory B cells.

In particular, T follicular regulatory cells (Tfr) play a crucial role in modulating B cell selection and affinity maturation within the germinal center (GC) microenvironment, which is relevant to the development of anti-drug-antibodies (ADA) (36). Although many of the mechanisms by which the Tfr regulate the selection process remain to be defined, Tfr cells appear to promote the survival and expansion of B cells with improved affinity for the antigen (37). Thus, the presence or absence of regulatory T cell epitopes in therapeutic proteins [such as Tregitopes (16)], may influence the development of anti-drug antibodies. Identifying and classifying such epitopes in scaffold proteins may inform as to which of the unmodified scaffold protein sequences are less likely to drive ADA, while improving their utility in therapeutic applications.

Tregs (both peripheral and Tfr) are known to require lower antigen concentrations for activation compared to T helper cells. This is because Tregs apparently express T cell receptors (TCRs) with higher affinity for T cell epitope sequences in self-antigens and that have lower affinity for similar sequences in foreign antigens, which allows them to recognize and respond to low levels of self-antigens in the absence of inflammation (38–42). In contrast, T effector cells generally require higher antigen concentrations and co-stimulatory signals for activation, which allows them to respond to foreign antigens during an immune response.

An interesting outcome of this imbalance between Treg and T effector TCR sensitivity is that foreign T cell epitopes that are nearly identical to Treg epitopes that are already present in the human genome, may drive Treg responses (43–45). This observation about foreign antigens that contain self-like sequences contributed to the development of the JanusMatrix tool for defining T reg epitopes in silico. The JanusMatrix tool was developed to predict such T cell epitopes (in foreign proteins, and later, in self-proteins), based on conservation of their TCR-facing residues with similar HLA-binding T helper epitopes in the human genome. This tool is now used to identify potential tolerogenic and tolerated epitopes.

JanusMatrix divides the amino acids present in the linear sequence of T cell epitopes into two sets of amino acids to find matching but not identical epitopes in the human genome. As illustrated in **Supplemental Figure 3**, T cells interrogate the surface of the peptide-MHC complex using their TCR, but amino acid side chains that are involved in anchoring the peptide to the bottom of the HLA binding groove are invisible to the TCR. Taking advantage of the linear conformation of MHC- (or HLA-) bound T cell epitopes, Moise et al. separated the MHC-binding amino acids from the TCR-facing amino acids, computationally, enabling the prospective identification of T cell epitopes that were conserved with, but not identical to, human proteome self-epitopes (46). The tool facilitates the identification of potentially “immunogenic” and “tolerated” epitopes found in proteins, peptide drugs, and vaccines (47). The JanusMatrix tool was specifically developed to find putative T cell epitopes in the human proteome that are restricted by the same MHC as the input sequence, even when they do not exactly match the input sequence, if they present the same TCR facing residues to the T cell and thus have the potential to generate a cross-conserved immune response. No other tools that are currently available in public toolkits provide this unique search capability, to our knowledge.

JanusMatrix scores are calculated by determining how extensive the cross-conservation of each potential epitope in a peptide is with peptides from the human genome that bind to the same MHC. The JanusMatrix score of a sequence reflects the depth of T cell epitope conservation with the sequence within the human proteome, giving each individually cross-conserved T cell epitope equal weight and dividing by the number of epitopes that have the potential to be cross-conserved in the parent peptide. Higher JanusMatrix scores denote greater degrees of conservation with the human proteome. Following a series of retrospective studies of existing epitope databases, Moise et al. identified a JanusMatrix score threshold of three, as defining potentially tolerated epitope (46), while T cell epitopes with JanusMatrix scores greater than five, are more likely to be actively tolerogenic (activate natural regulatory T cell responses) (48, 49). Not all high-scoring JanusMatrix epitopes are tolerogenic *in vitro*. Additional *in vitro* studies are generally carried out to confirm that the epitopes actively suppress T effector responses. The prevalence of the cross-conserved human protein, and/or the fact that it is secreted, may also have an impact on tolerance. It is notable that JanusMatrix enabled the identification of Treg epitopes in immunoglobulin (50), Factor V (51), and others have identified Treg epitopes in heat shock proteins (52).

One of the first applications of the JanusMatrix tool was to assess the cross-reactivity of T cell epitopes between 151 human pathogens and host (human) proteome epitope sequences. Cross-conservation was found to be more common in commensal pathogens (e.g. Herpes Simplex Virus, HSV) and less common in pathogens that had not adapted to humans (53, 54). This led to the concept of ‘immune camouflage’ and suggested that pathogens may evolve to escape immune responses. Furthermore, the epitopes that were the target of the pathogen camouflage epitope appeared to be extensively cross-conserved within the human genome, a feature of T cell epitopes that induced active tolerance (55, 56). The tool has been used to identify Treg epitopes in human pathogens such as Hepatitis C virus (55), and avian influenza virus H7N9 (57).

JanusMatrix has also helped to define tolerogenic epitopes in immunoglobulin G (IgG) (50) and blood coagulation factor V (51). The JanusMatrix tool has also been used to evaluate neo-antigen epitopes in cancer genomes (58). Recent publications describing *in vitro* methods to confirm predicted T reg epitopes, see references (51, 59), make it possible to validate new Tregitopes that have been defined using specialized in silico tools.

Since the prevalence of human proteome cross-conserved T cell epitopes appears to be relevant to tolerance, at least for Tregitopes and monoclonal antibodies (60, 61), it seems timely to explore epitope cross-conservation in commonly used “non-antibody” scaffold proteins to determine if the balance of immunogenic and tolerogenic epitopes is correlated with clinical immunogenicity. Natural scaffold proteins also contain HLA DRB1-binding peptides that have been eluted from HLA molecules and published in online databases such as the “HLA ligand Atlas” [see IEDB (35) and the HLA Ligand Atlas (62)], making it possible to confirm in silico predictions using published data, as described above for MAPPS data., and illustrated here.

The integration of this type of in silico analysis and data from publicly available HLA ligand databases, and *in vitro* studies using the regulatory T cell bystander suppression assay (51) and studies of responding T cell phenotypes, may help to further elucidate the relationship between the potential for immunogenicity and the observed immunogenicity of scaffold proteins.

## *In silico* approach to assessing immunogenicity risk

### *In silico* assessment with epitope-mapping tools

**EpiMatrix** (and other epitope mapping tools) are used to identify putative T cell epitopes in protein sequences. EpiMatrix is based on the pocket profile method developed by Hammer et al. as originally described in (63) and has improved over 25 years of continuous use by EpiVax experts through refinement of the epitope prediction matrices (23). It is used to predict HLA ligands for thousands of HLA class I and class II alleles, covering up to 95% of global populations (64). Scores higher than positive 20 on a normalized scale (the EpiMatrix Immunogenicity Scale, **Supplemental Figure 1**) indicate significant potential for immunogenicity. Based on an assessment of 10,000 randomly generated protein sequences, EpiMatrix Scores are normalized on a per 1000 assessments scale so that the expected score of a randomly generated protein sequence of a given length is set to zero. Positive scores denote more putative T cell epitope content than random chance and negative scores denote fewer putative T cell epitopes than random chance. The average human genome protein score is negative nine (–9) on the scale, and the average secreted protein score is negative 23 (–23). Standard vaccine antigens score from positive 20 (influenza HA) to greater than 80 (Hepatitis B Virus Surface Antigen (HBVsAg)). (See **Supplemental data for additional details**).

### JanusMatrix used for human homology and tolerance

JanusMatrix, searches HLA/epitope complexes for a human-like outer contour (TCR face) that can be recognized by T cells trained on self-epitopes from other prevalent and circulating human proteins. Improvements to in silico immunogenicity risk predictions using JanusMatrix lead to better definition and discrimination between immunogenic and tolerated T cell epitopes (as described above). JanusMatrix thresholds have been defined for epitopes that are more likely to generate tolerogenic responses or immunogenic responses based on the extent of cross-conservation between a given T cell epitope and similar HLA-binding T cell epitopes in the human genome. For each positive HLA-binding score in the source protein, JanusMatrix calculates the number of like-binding, TCR conserved sequences in the human proteome. The JanusMatrix score represents ‘depth of coverage” or, essentially, how many like-binding epitopes with similar TCR facing residues are found in the human genome. High (>3) JanusMatrix scores indicate high self-epitope conservation. In general, even higher JanusMatrix scores (>5) are found for *regulatory* T cell epitopes from IgG (65) and Factor V (51), and are rare. While higher JanusMatrix scores are correlated with tolerance *in vivo* (46, 51, 55, 57, 66, 67), EpiVax has also observed that some T cell epitopes that are conserved with self (but have JanusMatrix scores less than 3) are immunogenic. There are clearly aspects of “cross-conservation with self” that remain to be defined before JanusMatrix can be highly accurate, such as defining whether the prevalence of the peptide sequence or the protein that contains it is relevant to its tolerogenicity. Thus, JanusMatrix scores can be taken as a guide, and putatively tolerogenic epitopes that have high JanusMatrix scores should be carefully evaluated *in vitro* for tolerogenic activity to better assess their true immunomodulatory potential.

#### Validation using *in vitro* - HLA binding

Binding assays (68) are used as a second orthogonal (independent) method in addition to the in silico predictions to assess the HLA binding potential of a given epitope sequence. EpiVax performs soluble HLA DRB1 binding assays using typically seven to nine HLA DRB1 class II alleles that cover >85% of the human population (30) (see **Supplemental information** for discussion of the HLA Class II supertype alleles). HLA binding assays provide important validation of the HLA binding, although the phenotype of the immune response usually is determined using two types of T cell assays, described below.

#### *In vitro* assays to evaluate T cell response

T cell response (measured *in vitro*) is another independent method of assessment and is generally combined with HLA binding results to further support *in silico* predictions. Most biotech groups are using *in vitro* confirmation of immunogenicity to evaluate their candidate molecules (69). *In vitro* T cell assays use peptides representing predicted epitopes. T cell assays provide information about the ability of defined sequences to drive a T cell response (including an off-target immune response) and may be able to define the type of immune response elicited by the T cell epitope (effector or inflammatory, null, or regulatory). This is an essential step in the epitope validation process because *in silico* epitope prediction and *in vitro* HLA binding are not sufficient on their own to confirm T cell response.

#### (A) *In vitro* T cell assay (*in vitro* immunogenicity protocol)

Over the past 10 years, therapeutic protein developers have adapted an *in vitro* immunogenicity (IVIP) protocol from Wullner et al. (70) to test T cell response to biologics, using human PBMC. Blood from HLA-typed healthy individuals can be obtained from local blood banks. B cells, T cells, and antigen-presenting cells are separated from red blood cells for use in the assay. Epitopes are incubated with the cells to drive T cell responses. In general, overnight incubation is all that is required to generate a T cell response that is due to prior exposure (memory T cell response), while naïve T cell response can be generated in ten to 14 days. ELISpot assays are used to confirm the presence of responding T cells and flow cytometry assays can also be used, to better define the regulatory and effector T cell responses. Alternative (orthogonal) assays include dendritic cell assays developed by Laurent Malherbe (71) and MAPPS assays (72).

#### (B) Treg assay (Tetanus Toxoid Bystander Suppression Assay)

To evaluate the regulatory potential of highly cross-conserved epitope peptides, our group has adapted a previously published Tetanus Toxoid Bystander Suppression Assay (TTBSA) that measures the inhibitory capacity of potential regulatory peptides on the recall response of human CD4 T cells to the tetanus toxoid (TT) antigen (51, 73). We have set the TT-induced response (proliferation of memory CD4 T cells) in healthy donors at >10%, and suppression of response by the putative regulatory peptide must be statistically significant when compared to response to the TT antigen alone. These assays have been used to validate JanusMatrix predicted Treg epitopes. For example, we selected 22 peptides from GAA (the enzyme that is deficient in Pompe disease patients) with high JanusMatrix scores. The TTBSA confirmed that 4 of the 22 peptides were validated Treg peptides demonstrating greater than 40% suppression in the TTBSA (59). This assay has been used to define additional T cell epitopes that are regulatory in nature including some that are derived from human proteins such as IgG, coagulation factor V and alpha glucosidase (GAA) (51, 59). The assay was used to confirm Treg epitopes in the NSP7 protein of SARS-COV-2 (74) and avian influenza virus H7N9 (57).

### Potential immunogenicity and tolerogenicity of scaffold proteins

#### *In Silico* risk assessment of non-antibody scaffold proteins

To our knowledge, no in silico studies have been published for non-antibody scaffold proteins. Since many of these products are now approaching clinical use, it may be of interest to report the in silico risk assessment of these sequences, especially as some non-antibody scaffolds are human in origin and some are derived from bacterial proteins.

We therefore performed an Epi Matrix and JanusMatrix evaluation of the 12 scaffold proteins listed in **Table 1**. The protein sequences identified in the original publications (see **Table 1** for citations) were uploaded to the ISPRI toolkit. Methods are described in **Supplemental Material, Part 1**. EpiMatrix was used to parse each of the proteins into overlapping nine-mer frames for evaluation of HLA binding potential. Each nine-mer frame was evaluated for potential binding to one of nine HLA Class II alleles using the EpiMatrix algorithm, and the overall likelihood of binding was summed for each of the proteins and normalized on the EpiMatrix immunogenicity scale (**Supplemental Figure 1**), for comparison across different length proteins. ClustiMer was used to identify high-density clusters of predicted T cell epitopes that might generate a promiscuous T cell response (67). (See **Supplemental Figure 2**).

**Figure 2:**
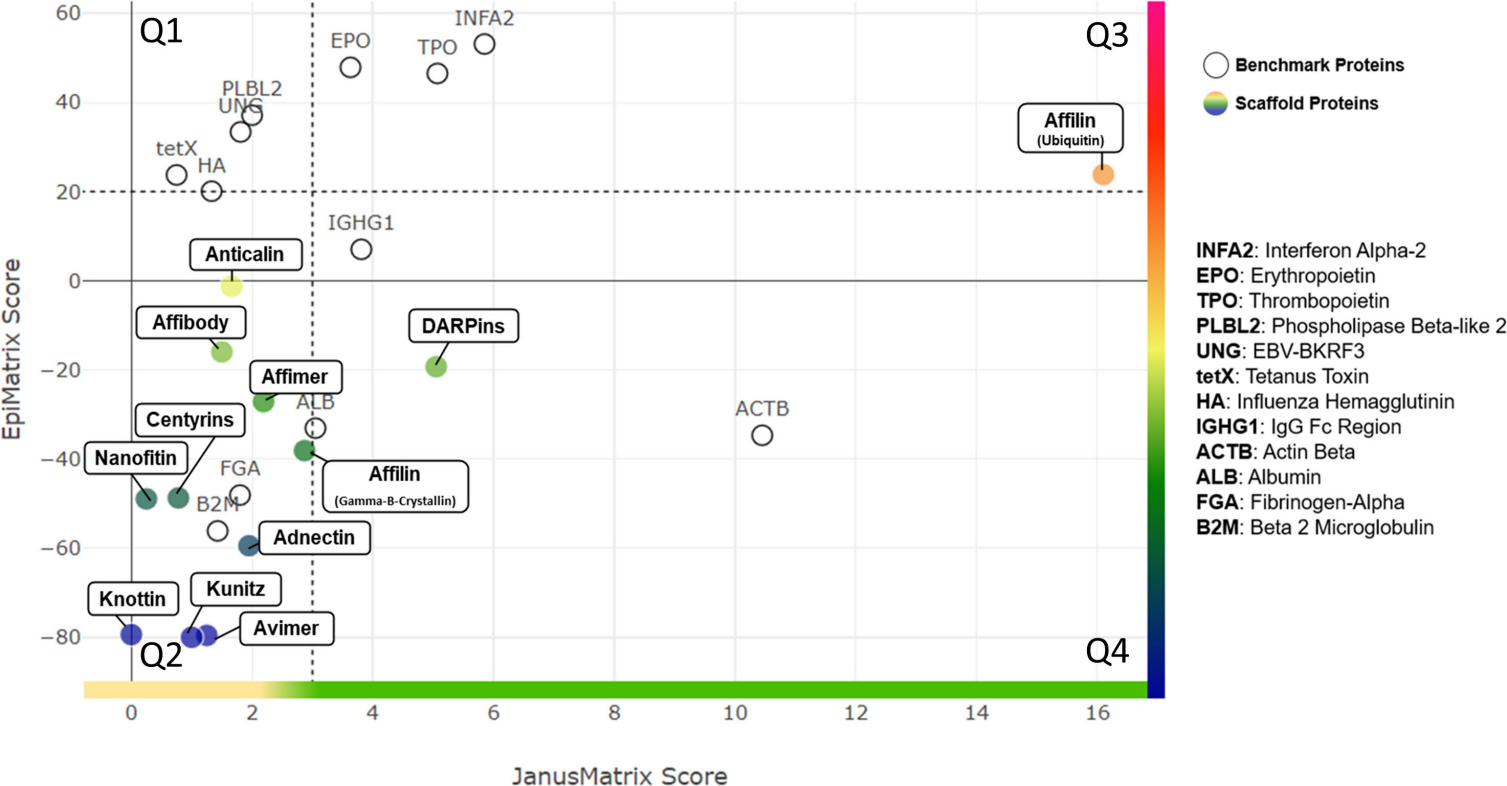
Quadrant Plot Analysis. EpiMatrix Protein Score (measure of epitope content) and JanusMatrix Protein Score (measure of humanness) are plotted for each scaffold protein. The Y-axis represents total epitope content or immunogenic potential. The JanusMatrix Score of a given protein indicates the average depth of coverage (in the human genome) for the HLA-binding peptides contained within that sequence. High JanusMatrix scores indicate high ‘Humanness” and are shown on the X axis. The plot is divided into quadrants that enable the categorization of proteins into one of four broad potential immunogenicity risk quadrants: (Q1) Highest Risk - Epitope Dense, Less Common in Human Proteins; (Q2) Moderate to High Risk: Epitope Sparse, Less Common in Human Proteins; (Q3) Moderate to Low Risk: Epitope Dense, More Common in Human Proteins; and (Q4) Lowest Risk: Epitope Sparse, More Common in Human Proteins. A set of proteins with known immunogenicity profiles are shown on the quadrant plot for comparison.

These clusters were then compared to the human genome and the degree of cross-conservation with self-epitopes was defined using JanusMatrix (**Supplemental Figure 3**). A whole protein JanusMatrix score was generated by summing the tolerogenic potential of self-like conserved T cell epitopes over the length of the entire protein. The EpiMatrix and JanusMatrix scores for each of these scaffold proteins are listed in **Table 2** and illustrated in **Figure 2**. The number of potentially immunogenic clusters (JanusMatrix less than 3) and potentially tolerogenic clusters (JanusMatrix greater than 5) is also noted for each protein.

**Table 2:** In Silico Analysis of 12 Scaffold Proteins. Total # of EpiMatrix Hits is the number of predicted HLA DRB1 ligands or T cell epitopes within the full length of the scaffold sequence.

COMMON NAME	SOURCE ORGANISM	PROTEIN	AA LENGTH	Total # of EpiMatrix Hits	EpiMatrix Protein Score	JanusMatrix Protein Score	Total # of T cell Epitope Clusters	# of “non-immunogenic” clusters (JMX > 3.0)	# of potentially tolerogenic clusters (JMX > 5.0)
**Anticalin**	Human	Lipocalin	178	80	-1.25	1.67	5	0	0
**Affilin**	Human	Gamma-B-Crystallin	175	55	-38.12	2.89	2	**1**	0
**DARPin**	Human	Ankyrin repeat proteins	166	65	-19.30	**5.06**	5	**2**	**2**
**Centyrin**	Human	Fibronectin Type III domain	103	27	-48.73	0.74	1	**1**	0
**Affimer**	Human	Cystatin-A	98	32	-27.13	2.19	2	0	0
**Adnectin**	Human	Fibronectin	94	21	-59.46	1.95	1	0	0
**Affilin**	Human	Ubiquitin	76	39	23.79	**16.10**	2	0	**2**
**Nanofitin/affitin**	Bacteria	DNA-Binding Sac7d	65	16	-48.95	0.25	1	0	0
**Affibody**	Bacteria	IgG-binding protein A	58	22	-15.98	1.50	1	0	0
**Kunitz**	Human	Collagen Alpha-3 VI-Chain	58	4	-93.77	1.00	0	0	0
**Avimer**	Human	Low-density lipoprotein receptor	37	4	-79.57	1.25	0	0	0
**Knottin**	Plant	Trypsin Inhibitor 2	28	3	-79.39	0.00	0	0	0
REFERENCE CONTROLS									
**Average Score of Random Proteins**					0.00				
**Median Score of Human Proteome**					-9.05				
**Median Score of Secreted Human Proteome**					-23.08				

EpiMatrix Protein Scores represent a measure of T cell immunogenicity potential for a given sequence and is the excess (positive scores) or shortfall (negative scores) in aggregate immunogenicity potential relative to a random protein sequence (zero). EpiMatrix Protein Scores are normalized on a per 1000 9-mer assessments scale to facilitate comparison of different length sequences. For all of the predicted epitope content within the full sequence of the given scaffold, JanusMatrix Protein Scores represent the average depth of cross-conservation with compatible (predicted to bind to same HLA) epitopes with exactly matching TCR-facing residues derived from the human proteome. T cell Epitope Clusters represent short regions (9-25 amino acids in length) of concentrated epitope density within the full scaffold sequences. T cell epitope clusters with JanusMatrix Scores >3.00 have an elevated potential for homology-induced tolerance and are potentially non-immunogenic. T cell epitope clusters with JanusMatrix Scores >5.00 have a significant potential of homology-induced tolerance and are potentially tolerogenic. Reference Controls indicate average or median EpiMatrix Protein Scores for sets of proteins. The EpiMatrix immunogenicity scale is set to zero based on the median HLA DRB1 score of a set of random protein sequences. The median score of all proteins in the human proteome is -9.05 while the median score of the secreted human proteins subset is even lower at -23.08.

Bold values represent proteins with a JanusMatrix score >5.00.

While this is far from a comprehensive list of scaffold proteins, and the analysis that has been performed still needs to be examined in the context of clinical studies (when those are available), the information provided illustrates the concept that non-antibody scaffold proteins, like immunoglobulins, contain T cell epitope clusters in their backbone sequences that are likely to have an impact on the immunogenicity risk of drug candidates derived from these proteins in the clinic.

As illustrated in **Table 2**, the backbone sequence of non-antibody scaffolds Anticalin, Affilin (Gamma-B-Crystallin), DARPins, Centyrins, Affimers and Adnectins had high overall numbers of T cell epitope “hits” as well as epitope clusters, although these clusters were distributed over large sequence space resulting in low overall EpiMatrix protein scores. Several epitope clusters were highly homologous with human epitopes (JanusMatrix score >5) in the case of Affilin (ubiquitin), DARPin, and slightly less so for Anticalin and Centyrin (JanusMatrix score >3). The human ubiquitin-based Affilin is striking for its higher overall T cell epitope content, which might otherwise indicate a high risk of immunogenicity, if not for the fact that the protein sequence contains quite a few clusters that are extensively conserved within the human genome.

The bacterial-origin scaffold nanofitin and affibody proteins had more limited cross-conservation with the human genome as demonstrated by their low JanusMatrix scores. Avimer and Knottin are notable for the near absence of T cell epitope ‘hits’, resulting in extremely low scores, which lower their potential for immunogenic response, even though their cross-conservation with the human genome is limited.

A more detailed discussion of the immunogenicity assessment for each of the individual scaffold proteins follows in the **Supplemental Information, Part 2**. While the overall potential of each of these proteins can be estimated from their overall EpiMatrix score and their overall JanusMatrix score, there is no combined EpiMatrix and JanusMatrix score that can be generated for these proteins, as methods for combining the scores have not yet been validated in prospective studies. Rather than combining the scores, it is possible to visualize the combined scores on a quadrant plot as illustrated in **Figure 2**.

## Discussion

In answer to the question posed in the title of this perspective, human homology may well reduce the potential immunogenicity of selected scaffolds, especially in the case of DARPins and ubiquitin-based Affillin. In addition, most of these non-antibody scaffold proteins have fewer T cell epitopes than the average human protein, which further reduces their potential to drive B cell response and anti-drug antibodies. However, the introduction of novel epitopes in the engineered domains may drive new T cell responses, leading to the development of ADA.

It is important to note that the intended mechanism of action of the scaffold protein therapeutic may have an impact on immunogenicity. At steady states, Treg cells require lower amounts of MHC-bound T cell epitopes to be activated, due to their higher affinity TCR, as compared to conventional T cells that may be specific for the same MHC-peptide complex (75). However, in the context of either acute or chronic inflammatory conditions or an environment in which pro-inflammatory cytokine levels are elevated (such as can be found in autoimmune diseases, or induced by check-point inhibitors such as those used in cancer therapy), conventional effector T cells can be activated and overcome the suppression by Tregs (76, 77).

Therefore, under certain inflammatory conditions, the countervailing balance of tolerogenic epitopes may be overcome, which will increase the potential immunogenicity of scaffold proteins that contain such epitopes. That is exactly why it is even more crucial to validate the presence of putatively tolerogenic epitopes in scaffold proteins, and to define whether the candidate will be used in a clinical context that risks induction of unexpected immunogenicity.

There are additional factors that can impact observed immunogenicity and lead to deviation from predicted values. These additional factors include differences in formulation, the presence or absence of impurities and/or host cell proteins, differences in dose, route of administration, target, mechanism of action, and the immune status of the patient population. The observed incidence of antibody (including neutralizing antibody) positivity in an assay may be influenced by several other factors including assay methodology, sample handling, timing of sample collection, concomitant medications, and underlying disease.

The degree to which any individual being treated with a therapeutic such as a scaffold protein may react to any of the T cell epitopes present in that therapeutic protein will also be dependent on which T cell epitopes are presented by their HLA, and whether those epitopes are cross-reactive to self-epitopes that are also restricted by the same HLA. This concept is discussed at greater length in an analysis of Infantile Pompe Disease subjects’ anti-therapeutic antibody responses to the replacement enzyme GAA. That article describes a method for assessing immunogenicity risk for individual patients by estimating the regulatory to effector ratio of T cell epitopes defined in the ERT as compared to their own residual GAA gene (59). Thus, future investigations into the immunogenicity risk of any therapeutic protein in an individual patient may benefit from approaches that can assess ‘personalized’ immunogenicity risk.

These discoveries are in line with research by other groups, in which the concept of the yin-and-yang balance of Treg and Teffector (or T helper) cells that recognize the same antigen is being identified [see excellent review by Santambroglio (78)]. There is now sufficient evidence to suggest that the peripheral T cell repertoire includes T cells with different phenotypes and different TCRs, that recognize the same MHC-self peptide complex, resulting in opposite immunological outcomes. When immune homeostasis is present, tolerance results. However, under inflammatory conditions associated with increased T cell activation, changes in the balance of signals can tilt the immune response towards the expansion of pro-inflammatory T cells.

Further studies will help elucidate these hypotheses, and much is to be learned from the experiments being conducted by non-antibody scaffold protein developers, as these exciting new molecules begin to enter clinical development.

## Data availability statement

All relevant data generated for this study are included in this article (please see **Supplemental Material** for additional details on methods and detailed scores). The raw data supporting the conclusions of this article will be made available by the authors to any qualified researcher upon request.

## Author contributions

ADG, AM, and WM conceptualized and designed the immunogenicity assessment of the non-antibody scaffold proteins. In silico implementation of algorithms were performed by AM, SK, and SL. All authors were involved in data analysis and also participated in drafting or revising the manuscript. All authors approved the submitted version.
