# Supplementary material for: Does human homology reduce the potential immunogenicity of non-antibody scaffolds?
Source: Front Immunol. 2023 Nov 7;14:1215939. doi: 10.3389/fimmu.2023.1215939 (PMC10664710; doi:10.3389/fimmu.2023.1215939)
Supplement: Supplementary file 1 [file DataSheet_1.pdf]

Supplemental Information for:

Does Human Homology Reduce the Potential Immunogenicity of Non-Antibody Scaffolds?

Anne S. De Groot , Sundos Khan, Aimee E. Mattei, Sandra Lelias, William D. Martin

Part 1. Description of the immunogenicity risk analysis approach, including EpiMatrix and JanusMatrix tools, used by EpiVax

A. EpiMatrix for enumerating putative HLA-ligand / T cell epitopes per unit length.

How EpiMatrix is used to identify potential T cell epitopes in protein sequences. To conduct an EpiMatrix analysis, input sequences are segmented into overlapping 9-mer frames and evaluated against a set of nine HLA-DR supertype alleles (See HLA DR selection criteria below). The primary objective of this assessment is to identify potential HLA class II-binding peptides that can elicit an immune response from T-helper cells. Each overlapping frame within the protein sequence is compared to a pre-defined matrix of HLA binding preferences for each of the nine supertype HLA-DR alleles. A score is assigned to each frame-allele assessment, indicating the likelihood of HLA-DR binding in comparison to a collection of previously published peptides found in the literature (for a description of EpiMatrix matrix development see references (1–3)).

The EpiMatrix assessment scores are standardized for each HLA-DR allele and, as a result, range from approximately -3 to +3. Scores exceeding 1.64 are considered "hits," suggesting a substantial probability of peptide binding to HLA molecules with moderate to high affinity. Consequently, these peptides are likely to be presented on the surface of antigen-presenting cells (APCs) such as dendritic cells or macrophages, potentially exposing them to passing T cells. Typically, around 5% of 9-mer peptides are anticipated to score above 1.64. (See early iVAX publication for details: Ref (3)).

B. Selecting Representative HLA DR alleles to cover the global population

HLA DR supertypes are a classification system that groups together similar human leukocyte antigen (HLA) DR alleles based on their MHC ligand specificities. These supertypes are selected based on the shared structural features (in the floor of the MHC-ligand binding groove) and overlapping peptide-binding motifs (specific amino acid side chains in the MHC ligands that bind into pockets in the binding groove) among different HLA DR alleles. The “binding motif” concept was first described by Rötzschke and Falk (4). By grouping HLA DR alleles into supertypes, it becomes possible to make broader predictions about antigen presentation and immune responses in global populations.

Since HLA molecules can be grouped into “supertypes”, a set of HLA DR supertypes can be identified to represent HLA DR from a substantial portion of the global populations. Approximately 95% of the human population can be classified into just nine major HLA DR Supertypes using known clustering patterns defined by Lund and Nielson(5,6) while also applying population coverage statistics developed by Bui et al.(7) and made available on the Immune Epitope Database (IEDB).

C. Quantifying Immunogenic Potentials: Immunogenicity Scales

All extracellular proteins including therapeutic proteins may be taken up by antigen-presenting cells (APCs) and processed in their Class II presentation pathway. Among the sequences that are processed are peptides that can bind to HLA DR (HLA ligands). These ligands may be displayed on the surface of the processing APCs, making them susceptible to immune surveillance by T helper cells. When all other factors are equal, a peptide or protein containing a higher number of HLA ligands or 'hits' is more likely to trigger an immune response by a higher number of T cells. To represent this idea, we have devised an immunogenicity scale incorporating EpiMatrix scores.

Supplemental Figure 1. EpiMatrix Scale

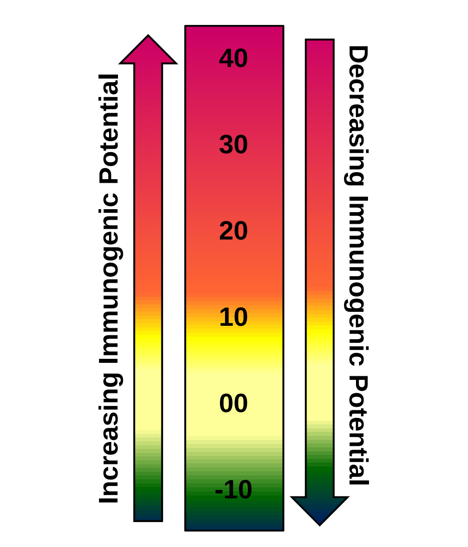

EpiMatrix Scores indicate potential for promiscuous immune response to proteins and peptides. Scores >20 and >10 are considered significant for proteins and peptides, respectively.

EpiMatrix Immunogenicity Scale. The EpiMatrix Score on the immunogenicity scale represents the difference between the number of predicted T cell epitopes one would expect to find in a randomly generated peptide or protein of a given size and the number of putative epitopes predicted by the EpiMatrix System for the protein or therapeutic being evaluated.

Since HLA DR scoring is normalized for each of the nine supertype alleles, EpiMatrix Scores can also be directly compared to each other, and the total putative epitope count can be plotted on a standardized scale (describing hits per 1000 amino acids).

The EpiMatrix Score of an "average" protein (represented by averaging the EpiMatrix protein scores of 10,000 randomly generated proteins) is set to zero on the immunogenicity scale. EpiMatrix Scores *above zero* indicate the presence of excess HLA ligands (as compared to random proteins) and denote a higher potential for immunogenicity, while scores *below zero* indicate the presence of fewer potential HLA ligands than expected, and a lower potential for immunogenicity than 'random'.

For most antigens, EpiMatrix Scores above +20 are generally considered to indicate significant immunogenic potential (**Supplemental Figure 1**). This is a threshold defined by reviewing (retrospectively) many published antigens.

However, human proteins generally score lower than zero, and secreted (circulating human proteins) score, on average, minus 23. In addition, some proteins scoring below zero on the scale can contain regions that are highly immunogenic. Peptides are evaluated on a separate scale that is set to an average peptide length, where scores greater than 10 are considered to have significant immunogenic potential. A detailed description of the immunogenicity scale and regional analysis of proteins is provided in the following publications on the iVAX toolkit (3,8).

D. Promiscuous Epitope-bars or EpiBars

Many of the most reactive proteins that induce a T cell response contain a feature referred to as an epitope-bar or "EpiBar". An EpiBar is a single nine-mer frame, which is predicted to bind to at

least four different HLA alleles (and is likely to be a promiscuous epitope). Sequences that contain epitope-bars include the well-known T cell antigens Influenza Hemagglutinin 306-318 (EpiMatrix Score of 22), Tetanus Toxin 825-850 (EpiMatrix Score of 46), and GAD65 557-567 (EpiMatrix Score of 23). An example of a well-known promiscuous epitope bar is shown below in **Supplemental Figure 2**. Sequences that contain EpiBars have been observed to bind to a range of HLA Class II molecules and tend to be very immunogenic in assays of blood samples drawn from human subjects.

Supplemental Figure 2. Example of an EpiBar

Example EpiBar

Accession: Influenza – Sequence: HA306-318

Frame Start	AA Sequence	Frame Stop	Hydro-phobicity	DRB1*0101 Z-Score	DRB1*0301 Z-Score	DRB1*0401 Z-Score	DRB1*0701 Z-Score	DRB1*0801 Z-Score	DRB1*0901 Z-Score	DRB1*1101 Z-Score	DRB1*1301 Z-Score	DRB1*1501 Z-Score	Hits
1	PRYVKQNTL	9	-0.26	0.68	0.46	-0.25	1.45	-0.16	1.15	0.44	-0.11	0.34	0
2	RYVKQNTLK	10	-0.32	-0.34	0.32	-0.1	-0.51	0.58	-0.77	-0.57	0.77	-0.23	0
3	YVKQNTLKL	11	-0.56	3.06	2.28	3.18	2.81	2.43	1.87	2.81	3.11	2.55	9
4	VKQNTLKLA	12	-0.05	0.97	1.51	0.95	1.06	1.62	1.12	2.01	1.7	1.41	2
5	KQNTLKLAT	13	-0.16	0.49	-0.1	0.22	0.54	1	1.36	0.89	0.86	1.34	0

Total Assessments Performed: 45	Hydrophobicity:-0.77	EpiMatrix Score (w/o flanks): 23.18
Top 10% of random peptides (not significant, near miss)	Top 5% of random peptides (significant hit)	Top 1% of random peptides (significant hit, highly likely)

Supplemental Figure 2. EpiMatrix analysis of a promiscuous influenza epitope. *The influenza HA peptide is an epitope known to be promiscuously immunogenic. It scores extremely high for all 9 alleles in EpiMatrix. Its cluster score is 23. Cluster scores higher than 10 are considered to be significant. The band-like EpiBar pattern is characteristic of promiscuous epitopes.*

Peptides containing promiscuously binding epitope-bars can be very powerful immunogens. For example, 100% of subjects exposed to either Tularemia or Vaccinia responded to T cell epitope pools containing between 20 and 50 promiscuous epitopes (9,10). Thus, the presence of one or more dominant T cell epitope clusters can potentially enable a significant anti-therapeutic immune response even in otherwise low-scoring peptides or proteins.

E. JanusMatrix to define homology with the human proteome

T cell epitopes which are homologues to common human proteins may be tolerated or even tolerogenic in normal human subjects. To identify specific homologies that may reduce immunogenic potentials, EpiVax developed the JanusMatrix algorithm.

Within any given T cell epitope there are certain amino acids that contact and bind with the HLA molecule and certain amino acids that contact the T cell receptor (TCR) of responding T cells. For Class II restricted epitopes, relative positions 1, 4, 6, and 9 contact the HLA and positions 2, 3, 5, 7, and 8 are available to the TCR. Pairs of potential T-cell epitopes with compatible, but not exactly matched, HLA binding anchors and exactly matched TCR-facing contours may be cross-reactive. In other words, CD4+ T cells engaged and activated by a given T cell-epitope may also be engaged and activated by a TCR epitope-matched homologue. For any given T cell epitope, the JanusMatrix algorithm can be used to identify potentially cross-reactive epitopes present in a reference database, in this case, the human proteome (11).

As depicted in **Supplemental Figure 3**, for a given 9-mer epitope, the JanusMatrix algorithm searches a reference database, considering the amino acid content of both the HLA-facing agretope and the TCR-facing epitope. Reference sequences with a compatible agretope (i.e., one that is predicted by EpiMatrix to bind the same HLA as the input peptide) and exactly matching the TCR contact residues of the input peptide are returned. The JanusMatrix Homology Score of a given peptide or protein indicates the average depth of coverage within the reference database for the HLA binding peptides contained within that sequence. When comparing peptide epitopes to the human proteome, JanusMatrix Human Homology Scores above two indicate an elevated level of conservation between the TCR-facing features of the input peptide or protein, and the TCR-facing features of proteins resident within the human genome. Scores above four are considered significant.

For human proteins, those thresholds are extended to three and five, because the human proteome reference database contains a copy of the input sequence. For a given EpiMatrix Score, a high JanusMatrix Human Homology Score suggests a bias towards immune tolerance. In other words, high JanusMatrix Human Homology Scores tend to offset high EpiMatrix Scores.

We have performed an analysis of 3,756 HLA Class II-restricted T cell epitopes for which cytokine release data are catalogued by the IEDB. This study indicates that there is a statistically significant relationship between high JanusMatrix Human Homology Scores and observed production of IL-10, a cytokine commonly associated with regulatory T cell responses, as well as a statistically significant inverse relationship between high JanusMatrix Human Homology Scores and observed production of IL-4, a cytokine commonly associated with inflammatory T cell responses (8).

EpiVax has shown that Tregitopes (actively tolerogenic HLA Class II T cell epitopes derived from human IgG and patented by EpiVax) are highly conserved not only among human antibodies, but also among other human proteins (11). In addition, EpiVax has demonstrated that certain pathogenic organisms have adapted and integrated human-like sequences as a form of immune camouflage. Thus, some HLA Class II T cell epitopes derived from influenza A virus and hepatitis C virus with high JanusMatrix Scores induce regulatory T cell responses (12,13).
In addition, EpiVax has shown that Tregitopes (actively tolerogenic HLA Class II T cell epitopes derived from human IgG and patented by EpiVax) are highly conserved not only among human antibodies, but also among other human proteins (10).The idea that natural regulatory T cells can be engaged and activated by peptide epitopes possessing TCR-facing motifs commonly found within human proteins has also been demonstrated in other studies (14).

Comparing putative HLA ligands to Known T cell epitopes or HLA ligands. To take advantage of any information regarding the phenotype of response to predicted ligands or their close relatives that may exist in the literature, potential T cell epitopes identified using epitope prediction tools can be screened against large databases such as the Immune Epitope Database of previously published HLA ligands and T cell epitopes and the HLA Ligandome atlas (15,16).

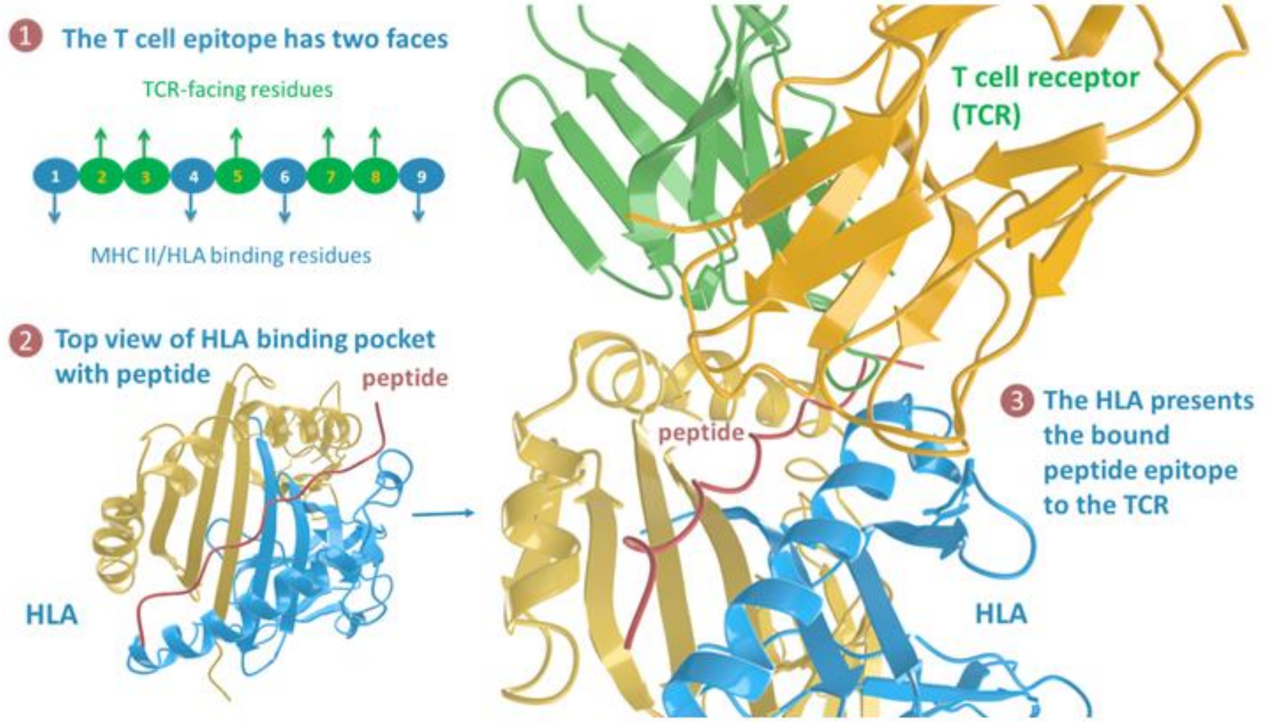

Supplemental Figure 3. Isolating TCR facing residues enables evaluation of epitope cross-conservation. ❶ The T cell peptide epitope has two faces. Amino acids in the binding pocket at positions 1, 4, 6 and 9 are facing the HLA molecule and are engaged in binding, where amino acids at positions 2, 3, 5, 7 and 8 are outwardly facing and are surveyed by a repertoire of the T cell receptors (TCR). ❷ Top view of the peptide epitope within the HLA binding pocket. ❸ Illustration of the MHC class II-peptide-TCR complex. The peptide epitope is bound to the HLA and presented at the surface of the antigen presenting cells to the T cell receptor (TCR). The TCR only sees the outward facing amino acids, and evaluating the homology of TCR-facing residues for cross-conservation to the human proteome is informative. The structures shown in graphic were adapted from NIH 3D entries ❷ 3DPX-002756 (17) and ❸ 3DPX-016438 (18). The different colored ribbons represent each polymer chain in their respective molecule. The pale yellow and blue ribbons correspond to the MHC class II structure. The gold and green ribbons represent the T cell receptor, and the red string represents the peptide epitope.

D. In vitro validation.

Most T cell epitopes can be validated using a combination of methods such as HLA Binding Assay and T cell assays. A competition-based HLA binding assay is used at EpiVax to evaluate peptide generic drugs and impurities; details are previously published (19). Alternatively, an In Vitro T cell assay (IVIP) can be employed to evaluate whether T cell responses can be generated to putative epitopes. This type of assay has also been published in detail (20). And finally, bystander suppression assays can be used to confirm the presence of regulatory T cell epitopes. The assay uses a common antigen (tetanus or another antigen to which most individuals have been exposed and for which a recall response would be expected in vitro) to establish a memory immune response. In parallel wells, a putative Treg epitope peptide is added to the assay, and changes in the cellular immune response to target ‘memory’ antigen can be measured either by EliSpot or Flow. A description of this assay and its application to the validation of a regulatory T cell (Treg) epitope from Factor V was recently published (De Groot, Rosenberg et al reference (21)).

Part 2. Detailed Immunogenicity of 12 Example Scaffold Protein Backbones

The development of drug products using these protein scaffolds depends on the introduction of mutations to 'variable domains' that target ligands or receptors into the natural scaffold protein sequence. Additional modifications may be made for process and production improvements. These modifications may introduce additional potentially immunogenic epitopes. Depending on the mechanism of action (discussed in greater detail below), the overall immunogenicity of the final product will depend on the impact of the new epitopes on the baseline potential for immunogenicity of the backbone sequence. A brief description of the baseline potential for immunogenicity of the natural, unmodified backbone of each of the 12 example scaffold proteins is provided in the next section.

Anticalins (22) are based on a naturally occurring protein called lipocalin. Lipocalin has a central barrel formed by eight antiparallel β-strands that is folded to form a pocket (23). The protein also features four structurally variable loops that connect the β-strands. These loops can be modified using random mutagenesis and molecular selection methods to create new molecules with high affinity binding capabilities. While initial clinical studies suggest that lipocalins have very low immunogenicity (24), more clinical data is needed.

The amino acid sequence of the anticalin analyzed here contains a significant number of predicted T cell epitopes (80 EpiMatrix "Hits"). Its EpiMatrix immunogenicity score (*minus* 1.2) is higher than the median score of human secreted proteins (-23). The JanusMatrix analysis suggests cross conservation (beyond natural lipocalins) with the human genome is limited. However HLA-DR restricted epitope clusters from lipocalins are frequently found in the HLA Ligand Atlas (25). The presence of significant numbers of predicted T cell epitopes and limited cross conservation with the human genome suggest that this scaffold protein may have some potential for immunogenicity in the clinic.

Two types of **Affilins** are included in Table 1 (main manuscript). One is derived from the sequence of human gamma-B-crystallin, a highly stable molecule that is expressed in the lens of the eye. Molecular engineering of eight solvent-exposed amino acid residues has enabled the development of a wide range of molecules. The backbone sequence of the gamma-B-crystallin Affilin contains 55 EpiMatrix hits. This is a significant number, but still fewer than we would expect to find in a protein of this size, and lower than the average number of epitopes found in secreted proteins in the human genome (average score EpiMatrix of proteins in the human secretome, which is *minus* 23, see Table 2, main manuscript). The EpiMatrix score of the gamma-B-crystallin Affilin is -38.12. The JanusMatrix score of this sequence (2.89) falls below our threshold value for JanusMatrix (3.0) suggesting a low level of cross conservation with the human genome. The presence of significant numbers of predicted T cell epitopes and limited cross conservation with the human genome suggest that this scaffold protein may have some potential for immunogenicity in the clinic.

The ubiquitin version of Affilin has a much higher EpiMatrix score (23.79) however, most of the epitope clusters in the ubiquitin backbone are highly cross conserved in different versions of human ubiquitin, which is highly prevalent protein, as reflected by its high JanusMatrix score (16.10). This combination of scores puts ubiquitin in a special category of proteins that are high scoring but contain Tregitope-like sequences, such as immunoglobulin G (where Treg epitopes have been well defined (26)). The potential tolerogenicity of the ubiquitin clusters should be validated in vitro, using an assay similar to the Tetanus Toxoid bystander suppression assay

mentioned above. Affilins are still in the early stages of development and little information on preclinical or clinical immunogenicity has been published.

DARPins (Designed Ankyrin Repeat Proteins) are based on naturally occurring ankyrin repeat domains (27). The DARPin sequence analyzed here contains 65 EpiMatrix hits. Although a significant number, this count falls significantly short of our expectation for a protein of this size (the average score for a protein of this size would be zero on the immunogenicity scale). Its EpiMatrix score (*minus* 19.3) is similar to scores observed among secreted human proteins (*minus* 23). Like ubiquitin-derived Affilins, DARPins also have extensive cross-conservation with the human genome (JanusMatrix score of 5.05) which may explain the low immunogenicity of these molecules in clinical use. For example, the pegylated DARPin drug abicipar (28), which is in clinical use for macular degeneration, has been reported to have low immunogenicity. It should be noted that the potential immunogenicity of the drug may be moderated by its use in the eye (an immunologically privileged site). However, intra-ocular inflammation (IOI) has been noted to occur after use of abicipar, and corticosteroids are used to reduce the inflammation. Notably, IOI is frequently associated with abicipar in clinical use (29). Since no anti-drug-antibody studies appear to have been performed, it is not clear whether the immunogenicity of this drug would be as high as was observed for the anti-VEGF DARPin drug (nearly 50% of subjects had ADA)(30). Some of this immunogenicity may be due to epitopes introduced in the targeting residues, or to the mechanism of action (induction of inflammatory responses), host cell proteins or process-related impurities.

Adnectins, **Monobodies**, and **Centyrins** are multimer (Adnectin) and monomer (Monobody, Centyrin) scaffold proteins, respectively, that are derived from the type III domain of human fibronectin. FNIII domains are arranged in a linear fashion and each domain unit is formed from two beta-sheets that are separated by an alpha-helix. This structure is stabilized by a disulfide bond. The C- and N-terminal domains of the fibronectin-derived scaffold proteins can be modified, which enables them to bind to target proteins. Fibronectin is a glycoprotein that plays a crucial role in cell adhesion, migration, and tissue organization within the extracellular matrix.

The **Adnectin** backbone analyzed here contains just 22 predicted T cell epitopes. At *minus* 59.46, its EpiMatrix protein score is extremely low. On the other hand, the JanusMatrix Score for the sequence (1.95) is also low, suggesting that there are few counterbalancing human-like epitope clusters in Adnectins that could reduce immune responses to the epitopes present in the backbone and any novel epitopes that are introduced in the variable loop domains. One of the earliest Adnectins to be developed, CT-322, progressed to Phase II before being discontinued due to adverse effects that were apparently unrelated to immunogenicity (31) . It is interesting, however, to note that 31 of 38 (82%) patients developed non-interfering antidrug antibodies to CT-322 (32). Competition binding with the human fibronectin-based wild-type Adnectin showed that the antidrug antibodies bound to the engineered binding loops of CT-322 (33). This finding would be in keeping with the observed in silico analysis, which finds low T cell epitope content but limited cross-conservation of T cell epitopes in Adnectins with other epitopes in the human genome.

Nanofitins are non-human scaffolds that are based on the DNA-binding protein of Sulfolobus acidocaldarius, an archaeal microorganism. The DNA binding protein structure is highly stable at very low pH and high temperatures (34). The Nanofitin sequence analyzed here contains a low number of predicted T cell epitopes (16) resulting in a very low EpiMatrix Score of *minus* 48.95. The low JanusMatrix Score of this protein (0.25) suggests that the few epitopes that are present may be recognized as foreign by the human immune system. When paired with novel epitopes

introduced during product development or when delivered into a pro-inflammatory environment these epitopes could induce or contribute to the development of an anti-drug immune response One nanofitin is in clinical development for treatment of complement-mediated disorders (35); no immunogenicity has been reported.

Affibodies are based on the non-human Protein A scaffold of Staphylococcus (Protein A binds immunoglobulin molecules). Protein A is well known to antibody developers and is known to be non-immunogenic in general. In silico analysis confirms that it has low T cell epitope content (22 EpiMatrix Hits and EpiMatrix score of *minus* 15.98) but only limited conservation with known human genome epitopes (JanusMatrix score of 1.50). Similar to the case of Nanofitins described above, when paired with novel epitopes introduced during product development or when delivered into a pro-inflammatory environment the few epitopes identified here could induce or contribute to the development of an anti-drug immune response.

Some of the smallest non-antibody scaffolds, the **Kunitz** scaffold, the **Avimer** scaffold, and the **Knottins** have very few potential T cell epitopes and extremely low EpiMatrix scores (*minus* 93.77, *minus* 79.57, and *minus* 79.39 respectively). One **Kunitz** protein scaffold is currently approved for human use and has not been reported to be immunogenic: it is Ecallantide, which is a specific inhibitor of plasma kallikrein. It was identified through phage display technology. No immunogenicity has been described, which is consistent with the low T cell epitope content of the sequence (EpiMatrix score of *minus* 93.77).

Avimers are a class of non-antibody scaffold proteins that are derived from avidin, although LDL receptor has also been used as a source. AVB-500 is a potential therapy for ovarian cancer, that targets GAS6. No immunogenicity has been described, which is consistent with the low EpiMatrix score (*minus* 79.57).

Knottins share a common structural motif, characterized by a core of antiparallel β-strands stabilized by at least three disulfide bonds. In a characteristic cystine-knot motif, the first and fourth and the second and fifth cysteine residues form disulfide bonds; the disulfide bond formed between the third and sixth cysteine residues passes through the other two disulfides, creating a macrocyclic knot (36). Their extremely low EpiMatrix score (*minus* 79.39) suggests that these small proteins may not be immunogenic in the clinic, however, modification and introduction of changes to the sequence may result in unexpected immunogenicity.

References for Supplemental Information

1. Schafer J. Prediction of well-conserved HIV-1 ligands using a matrix-based algorithm, EpiMatrix. *Vaccine* (1998) **16**:1880–1884. doi:10.1016/S0264-410X(98)00173-X

2. De Groot AS, Bishop EA, Khan B, Lally M, Marcon L, Franco J, Mayer KH, Carpenter CCJ, Martin W. Engineering immunogenic consensus T helper epitopes for a cross-clade HIV vaccine. *Methods* (2004) **34**:476–87. doi:10.1016/j.ymeth.2004.06.003

3. Moise L, Gutierrez A, Kibria F, Martin R, Tassone R, Liu R, Terry F, Martin B, De Groot AS. iVAX: An integrated toolkit for the selection and optimization of antigens and the design of epitope-driven vaccines. *Hum Vaccin Immunother* (2015) **11**:2312–2321. doi:10.1080/21645515.2015.1061159

4. Rötzschke O, Falk K. Origin, structure and motifs of naturally processed MHC class II ligands. *Curr Opin Immunol* (1994) **6**:45–51. doi:10.1016/0952-7915(94)90032-9

5. Lund O, Nielsen M, Kesmir C, Petersen AG, Røder G, Justesen S, Lundegaard C, Worning P, Sylvester-Hvid C, Lamberth K, et al. Definition of supertypes for HLA molecules using clustering of specificity matrices. *Immunogenetics* (2004) **55**:797–810. doi:10.1007/s00251-004-0647-4

6. Nielsen M, Lundegaard C, Blicher T, Peters B, Sette A, Justesen S, Buus S, Lund O. Quantitative predictions of peptide binding to any HLA-DR molecule of known sequence: NetMHCIIpan. *PLoS Comput Biol* (2008) **4**:e1000107. doi:10.1371/journal.pcbi.1000107

7. Bui H-H, Sidney J, Dinh K, Southwood S, Newman MJ, Sette A. Predicting population coverage of T-cell epitope-based diagnostics and vaccines. *BMC Bioinformatics* (2006) **7**:153. doi:10.1186/1471-2105-7-153

8. De Groot AS, Moise L, Terry F, Gutierrez AH, Hindocha P, Richard G, Hoft DF, Ross TM, Noe AR, Takahashi Y, et al. Better Epitope Discovery, Precision Immune Engineering, and Accelerated Vaccine Design Using Immunoinformatics Tools. *Front Immunol* (2020) **11**:442. doi:10.3389/fimmu.2020.00442

9. McMurry JA, Gregory SH, Moise L, Rivera D, Buus S, De Groot AS. Diversity of Francisella tularensis Schu4 antigens recognized by T lymphocytes after natural infections in humans: identification of candidate epitopes for inclusion in a rationally designed tularemia vaccine. *Vaccine* (2007) **25**:3179–91. doi:10.1016/j.vaccine.2007.01.039

10. Moise L, McMurry JA, Buus S, Frey S, Martin WD, De Groot AS. In silico-accelerated identification of conserved and immunogenic variola/vaccinia T-cell epitopes. *Vaccine* (2009) **27**:6471–6479. doi:10.1016/j.vaccine.2009.06.018

11. Moise L, Gutierrez AH, Bailey-Kellogg C, Terry F, Leng Q, Abdel Hady KM, VerBerkmoes NC, Sztein MB, Losikoff PT, Martin WD, et al. The two-faced T cell epitope: Examining the host-microbe interface with JanusMatrix. *Hum Vaccin Immunother* (2013) **9**:1577–1586. doi:10.4161/hv.24615

12. Liu R, Moise L, Tassone R, Gutierrez AH, Terry FE, Sangare K, Ardito MT, Martin WD, De Groot AS. H7N9 T-cell epitopes that mimic human sequences are less immunogenic and may induce Treg-mediated tolerance. *Hum Vaccin Immunother* (2015) **11**:2241–52. doi:10.1080/21645515.2015.1052197

13. Moise L, Terry F, Gutierrez AH, Tassone R, Losikoff P, Gregory SH, Bailey-Kellogg C,

Martin WD, De Groot AS. Smarter vaccine design will circumvent regulatory T cell-mediated evasion in chronic HIV and HCV infection. *Front Microbiol* (2014) **5**: doi:10.3389/fmicb.2014.00502

14. Hsieh L-E, Song J, Tremoulet AH, Burns JC, Franco A. Intravenous immunoglobulin induces IgG internalization by tolerogenic myeloid dendritic cells that secrete IL-10 and expand Fc-specific regulatory T cells. *Clin Exp Immunol* (2022) **208**:361–371. doi:10.1093/cei/uxac046

15. Marcu A, Bichmann L, Kuchenbecker L, Kowalewski DJ, Freudenmann LK, Backert L, Mühlenbruch L, Szolek A, Lübke M, Wagner P, et al. HLA Ligand Atlas: a benign reference of HLA-presented peptides to improve T-cell-based cancer immunotherapy. *J Immunother Cancer* (2021) **9**:e002071. doi:10.1136/jitc-2020-002071

16. Blazeska N, Kosaloglu-Yalcin Z, Vita R, Peters B, Sette A. “IEDB and CEDAR: Two Sibling Databases to Serve the Global Scientific Community,” in, 133–149. doi:10.1007/978-1-0716-3239-0_9

17. Murthy VL, Stern LJ. The class II MHC protein HLA-DR1 in complex with an endogenous peptide: implications for the structural basis of the specificity of peptide binding. *Structure* (1997) **5**:1385–1396. doi:10.1016/S0969-2126(97)00288-8

18. Deng L, Langley RJ, Brown PH, Xu G, Teng L, Wang Q, Gonzales MI, Callender GG, Nishimura MI, Topalian SL, et al. Structural basis for the recognition of mutant self by a tumor-specific, MHC class II–restricted T cell receptor. *Nat Immunol* (2007) **8**:398–408. doi:10.1038/ni1447

19. Kotraiah V, Phares TW, Terry FE, Hindocha P, Silk SE, Nielsen CM, Moise L, Tucker KD, Ashfield R, Martin WD, et al. Identification and Immune Assessment of T Cell Epitopes in Five Plasmodium falciparum Blood Stage Antigens to Facilitate Vaccine Candidate Selection and Optimization. *Front Immunol* (2021) **12**: doi:10.3389/fimmu.2021.690348

20. Wullner D, Zhou L, Bramhall E, Kuck A, Goletz TJ, Swanson S, Chirmule N, Jawa V. Considerations for optimization and validation of an in vitro PBMC derived T cell assay for immunogenicity prediction of biotherapeutics. *Clin Immunol* (2010) **137**:5–14. doi:10.1016/j.clim.2010.06.018

21. De Groot AS, Rosenberg A, Miah SMS, Skowron G, Roberts BJ, Lélias S, Terry FE, Martin WD. Identification of a potent regulatory T cell epitope in factor V that modulates CD4+ and CD8+ memory T cell responses. *Clin Immunol* (2021) **224**:108661. doi:10.1016/j.clim.2020.108661

22. Deuschle F-C, Ilyukhina E, Skerra A. Anticalin® proteins: from bench to bedside. *Expert Opin Biol Ther* (2021) **21**:509–518. doi:10.1080/14712598.2021.1839046

23. Flower DR. The lipocalin protein family: structure and function. *Biochem J* (1996) **318 (Pt 1**:1–14. doi:10.1042/bj3180001

24. Mross K, Richly H, Fischer R, Scharr D, Büchert M, Stern A, Gille H, Audoly LP, Scheulen ME. First-in-Human Phase I Study of PRS-050 (Angiocal), an Anticalin Targeting and Antagonizing VEGF-A, in Patients with Advanced Solid Tumors. *PLoS One* (2013) **8**:e83232. doi:10.1371/journal.pone.0083232

25. Marcu A, Bichmann L, Kuchenbecker L, Kowalewski DJ, Freudenmann LK, Backert L et al. The HLA Ligand Atlas - a resource of natural HLA ligands presented on benign tissues.

bioRxiv:778944v2 (2020) doi:10.1101/778944

26. De Groot AS, Moise L, McMurry JA, Wambre E, Van Overtvelt L, Moingeon P, Scott DW, Martin W. Activation of natural regulatory T cells by IgG Fc-derived peptide “Tregitopes”. *Blood* (2008) **112**:3303–11. doi:10.1182/blood-2008-02-138073

27. Stumpp MT, Dawson KM, Binz HK. Beyond Antibodies: The DARPin® Drug Platform. *BioDrugs* (2020) **34**:423–433. doi:10.1007/s40259-020-00429-8

28. Khurana RN, Kunimoto D, Yoon YH, Wykoff CC, Chang A, Maturi RK, Agostini H, Souied E, Chow DR, Lotery AJ, et al. Two-Year Results of the Phase 3 Randomized Controlled Study of Abicipar in Neovascular Age-Related Macular Degeneration. *Ophthalmology* (2021) **128**:1027–1038. doi:10.1016/j.ophtha.2020.11.017

29. Iyer PG, Albini TA. Drug-related adverse effects of antivascular endothelial growth factor agents. *Curr Opin Ophthalmol* (2021) **32**:191–197. doi:10.1097/ICU.0000000000000757

30. Baird RD, Linossi C, Middleton M, Lord S, Harris A, Rodón J, Zitt C, Fiedler U, Dawson KM, Leupin N, et al. First-in-Human Phase I Study of MP0250, a First-in-Class DARPin Drug Candidate Targeting VEGF and HGF, in Patients With Advanced Solid Tumors. *J Clin Oncol* (2021) **39**:145–154. doi:10.1200/JCO.20.00596

31. Schiff D, Kesari S, de Groot J, Mikkelsen T, Drappatz J, Coyle T, Fichtel L, Silver B, Walters I, Reardon D. Phase 2 study of CT-322, a targeted biologic inhibitor of VEGFR-2 based on a domain of human fibronectin, in recurrent glioblastoma. *Invest New Drugs* (2015) **33**:247–53. doi:10.1007/s10637-014-0186-2

32. Mamluk R, Carvajal IM, Morse BA, Wong H, Abramowitz J, Aslanian S, Lim A-C, Gokemeijer J, Storek MJ, Lee J, et al. Anti-tumor effect of CT-322 as an adnectin inhibitor of vascular endothelial growth factor receptor-2. *MAbs* (2010) **2**:199–208. doi:10.4161/mabs.2.2.11304

33. Tolcher AW, Sweeney CJ, Papadopoulos K, Patnaik A, Chiorean EG, Mita AC, Sankhala K, Furfine E, Gokemeijer J, Iacono L, et al. Phase I and pharmacokinetic study of CT-322 (BMS-844203), a targeted Adnectin inhibitor of VEGFR-2 based on a domain of human fibronectin. *Clin Cancer Res* (2011) **17**:363–71. doi:10.1158/1078-0432.CCR-10-1411

34. Gera N, Hussain M, Wright RC, Rao BM. Highly stable binding proteins derived from the hyperthermophilic Sso7d scaffold. *J Mol Biol* (2011) **409**:601–16. doi:10.1016/j.jmb.2011.04.020

35. Garlich J, Cinier M, Chevrel A, Perrocheau A, Eyerman DJ, Orme M, Kitten O, Scheibler L. Discovery of APL-1030, a Novel, High-Affinity Nanofitin Inhibitor of C3-Mediated Complement Activation. *Biomolecules* (2022) **12**: doi:10.3390/biom12030432

36. Schwalen CJ, Babu C, Phulera S, Hao Q, Wall D, Nettleton DO, Pathak TP, Siuti P. Scalable Biosynthetic Production of Knotted Peptides Enables ADME and Thermodynamic Folding Studies. *ACS omega* (2021) **6**:29555–29566. doi:10.1021/acsomega.1c03707
